# Indoor Heating Drives Water Bacterial Growth and Community Metabolic Profile Changes in Building Tap Pipes during the Winter Season

**DOI:** 10.3390/ijerph121013649

**Published:** 2015-10-27

**Authors:** Hai-Han Zhang, Sheng-Nan Chen, Ting-Lin Huang, Pan-Lu Shang, Xiao Yang, Wei-Xing Ma

**Affiliations:** School of Environmental and Municipal Engineering, Xi’an University of Architecture and Technology, Xi’an 710055, Shaanxi, China; E-Mails: chenshengnan@xauat.edu.cn (S.-N.C.); huangtinglin@xauat.edu.cn (T.-L.H.); shangpanlu@xauat.edu.cn (P.-L.S.); ichitake@126.com (X.Y.); hs_weixing@163.com (W.-X.M.)

**Keywords:** building indoor pipes, indoor heating, flow cytometry, BIOLOG

## Abstract

The growth of the bacterial community harbored in indoor drinking water taps is regulated by external environmental factors, such as indoor temperature. However, the effect of indoor heating on bacterial regrowth associated with indoor drinking water taps is poorly understood. In the present work, flow cytometry and community-level sole-carbon-source utilization techniques were combined to explore the effects of indoor heating on water bacterial cell concentrations and community carbon metabolic profiles in building tap pipes during the winter season. The results showed that the temperature of water stagnated overnight (“before”) in the indoor water pipes was 15–17 °C, and the water temperature decreased to 4–6 °C after flushing for 10 min (“flushed”). The highest bacterial cell number was observed in water stagnated overnight, and was 5–11 times higher than that of flushed water. Meanwhile, a significantly higher bacterial community metabolic activity (*AWCD*_590nm_) was also found in overnight stagnation water samples. The significant “flushed” and “taps” values indicated that the *AWCD*_590nm_, and bacterial cell number varied among the taps within the flushed group (*p* < 0.01). Heatmap fingerprints and principle component analyses (PCA) revealed a significant discrimination bacterial community functional metabolic profiles in the water stagnated overnight and flushed water. Serine, threonine, glucose-phosphate, ketobutyric acid, phenylethylamine, glycerol, putrescine were significantly used by “before” water samples. The results suggested that water stagnated at higher temperature should be treated before drinking because of bacterial regrowth. The data from this work provides useful information on reasonable utilization of drinking water after stagnation in indoor pipes during indoor heating periods.

## 1. Introduction

Building indoor water pipes are the most important drinking water facility related to potable water quality and public health [1,2]. In the past few decades, volatile organic compounds, heavy metal and pathogen contaminations in household pipes were routinely evaluated [3]. Compared with the numerous reports on bacterial cell concentration and community from drinking water reservoirs [4,5], drinking water treatment processes [6], and transportation system [7], the characteristics of water bacterial composition associated with building indoor pipelines remains poorly understood. Recent publications have suggested that drinking water pipeline materials, including metal pipes and polymer pipes [8,9], household stagnation [10] and temperature [3] may increase the bacterial regrowth associated with indoor water pipes [10].

Flow cytometry and the community-level sole-carbon-source utilization technique named BIOLOG have been combined to determine bacterial cell number and community metabolic fingerprints associated with drinking water [10] and drinking water reservoirs [4]. BIOLOG-ECO microplates contain 31 different sole carbon sources including carbohydrates, carboxylic acids, amino acids, polymers, phenolic compounds, and amines [4]. These carbon sources are utilized by water bacteria, and the metabolic profiles can be used to evaluate the community functional diversity [4]. Lautenschlager *et al.* [10] used flow cytometry and polymerase chain reaction and denaturing gradient gel electrophoresis (PCR-DGGE) methods to explore the effects of overnight stagnation on water bacterial growth and community genetic diversity in household taps, and suggested that short flushing of household taps prior to consumption should be carried out, because overnight stagnation significantly increased water bacterial growth [10]. However, the information on the bacterial community metabolic characteristics induced by overnight stagnation during the winter season with indoor heating is limited.

Indoor heating is the popular and traditional heating method used in cold areas, especially in northwest China [11]. Heating pipes are distributed in the walls of building facilities [11]. When the outdoor temperature drops below 10–20 °C, the indoor environmental temperature can reach 25–27 °C in a building after the municipal heating process occurs [12]. Biological stability in tap water is becoming a hot topic; Lee *et al.* demonstrated that higher indoor temperatures could improve water bacterial regrowth in drinking water pipes [3]. Similarly, Nam and Lee found that effluent water bacterial cell concentration was higher at 55 °C than that at 20 °C in polyethylene (PEX) pipe [13]. Unfortunately, no information is available about how indoor heating drives water bacterial regrowth and metabolic functional diversity in building tap pipes during winter [14].

Expanding our knowledge of bacterial regrowth and carbon metabolism characteristics in building indoor water pipes will improve our understanding of the biological stability of drinking water. Consequently, the main objective of this work is to explore how indoor heating drives water bacterial regrowth and community metabolic functional diversity in building tap pipes during the winter season in Xi’an City, Shaanxi Province, Northwest China. To this end, the specific objectives of this work are: (i) to determine the effects of the indoor heating process on water bacterial cell concentration; (ii) to examine water bacterial community functional metabolic profiles in different building tap pipes during the winter season.

## 2. Experimental Section

### 2.1. Sampling Description

This work was carried out in Xi’an City, Shaanxi Province, Northwest China (E107°40′–109°49′, N33°39′–34°45′). In Xi’an City, municipal central heating is one of the main methods of heating. Municipal heating is available from the middle of November to the middle of March of the following year [15]. Buildings with similar heating and indoor drinking water pipes were selected in this work. The material of the pipes is galvanized steel. In December 2014, when indoor temperatures were around 25 °C and the outdoor average temperature was below 1–4 °C, indoor tap water samples were individually collected after overnight stagnation from five buildings labelled building 1 (Tap 1), building 2 (Tap 2), building 3 (Tap 3), building 4 (Tap 4), and building 5 (Tap 5). Indoor air and tap water temperature were determined using a thermometer (Deli, Ningbo, China). According to the method described by Lautenschlager *et al.* [10] and with little modification, three individual taps (*n* = 3) ware randomly selected in each building for the sampling process [10]. In order to determine the effect of indoor heating on bacterial growth in water stagnated overnight in building tap pipes, the stagnation time of the sampling taps was more than seven hours. During the sampling process, the first liter of water (marked as “before”) was collected using a sterile flask (Corning, Biotechnology Co., Ltd, Shanghai, China), and after flushing 10 min, when the water temperature had decreased sharply, another liter of water was collected (marked as “flushed”). Then, the water samples were transported immediately to the Key Laboratory of Shaanxi Environmental Engineering, School of Environmental and Municipal Engineering, Xi’an University of Architecture and Technology (SEME, XAUAT, Xi’an, China). One part was used for water quality and water bacterial cell concentration determination, and another part was used for water bacterial community functional metabolic examination within 24 h after the sampling process [4].

### 2.2. Water Quality Determination

To examine the water quality of water stagnated overnight and flushed water samples, water temperature was determined by a thermometer (Deli, Ningbo, China). Water pH, dissolved organic carbon (DOC), ultraviolet absorbance at 254 nm (UV_254_), turbidity, and residual chlorine content were analyzed according to the procedures described by Huang *et al.* [16], Liang and Singer [17]. Water pH and turbidity were determined by a portable pH and turbidity meter (HACH, Loveland, CO, USA). DOC was measured using a TOC analyser (HACH) after first acidifying the samples [17].

### 2.3. Flow Cytometry Examination

To determine the water bacterial cell concentration, the flow cytometry technique was used. According to the method described by Nescerecka *et al.* [18] and Liu *et al.* [19], for cell staining process, working solutions including SYBR^®^ Green I and propidium iodide (PI) was mixed and stored at −20 °C until use [20]. Before determination, water samples were incubated for 15 min in the darkness. FCM was performed using a BD Accuri C6 System (BD Accuri cytometers, Aalst, Belgium) at a fixed wavelength of 488 nm [18,19,20]. FCM was measured in triplicate (*n* = 3).

### 2.4. Bacterial Community Metabolic Determination

To examine the water bacterial community functional metabolic fingerprints, the BIOLOG method was used [4]. There are 31 different carbon sources on the ECO microplate [4,21,22], including carbohydrates, carboxylic acids, amino acids, polymers, phenolic compounds, and amines (Table 1) [4,21,22]. BIOLOG is widely used to explore environmental microbial community functional diversity [4,21,22].

**Table 1:** Sole carbon sources used in the BIOLOG ECO microplate used in this work [4,21,22].

Carbohydrates	Carboxylic Acids	Amino Acids	Polymers	Phenolic Compounds	Amines
D,L-α-Glycerol phosphate	Pyruvic acid methyl ester	Arginine	α-Cyclodextrin Glycogen	4-Hydroxybenzoic acid	Phenylethyl-amine Putrescine
β-Methyl-D-glucoside	γ-Hydroxybutyric acid	Threonine	Tween40	2-Hydroxybenzoic acid	
i-Erythritol	D-Galacturonic acid	Serine	Tween80		
D-Cellobiose	α-Ketobutyric acid	Phenylalanine			
D-Mannitol	D-Glucosaminic acid	Asparagine			
D-Xylose	D-Malic acid	Glycyl-L-glutamic acid			
Glucose-1-phosphate	Itaconic acid				
N-Acetyl-D-glucosamine					
D-Galactonic acid γ-lactone					

There are three replicates on each ECO plate, and 96 wells in each plate. According to the method described in our previous study [4], briefly, in the clean bench, we added 150 μL water into each well of the ECO plate using an electronic pipette [4]. All inoculated BIOLOG micro plates were then incubated at 28 ± 2 °C in a dark chamber (Jinghong, Shanghai, China) for 10 days [4,21]. After 240 h incubation, the raw data (OD value) of each BIOLOG ECO plate were examined using an Elisa reader (BIOLOG Company, Hayward, CA, USA) at 590 nm [4,21]. In previous studies, 96 h or 144 h incubation data was used for the calculations [4,21]. However, in this work, the water samples were potable water, and the data of 240 h incubation was used for *AWCD*_590nm_, carbohydrates, carboxylic acids, amino acids, polymers, phenolic compounds, and amines metabolic, heat map and principle component analyses. *AWCD*_590nm_ represented the bacterial community metabolic activity [4,21], and was calculated using the formula described in Garland [21] and Zhang *et al.* [4].

### 2.5. Data Analyses

Data were recorded as the mean and standard errors (S.E) (*n* = 3), and analyzed by two-way ANOVA followed by Tukey-Kramer HSD tests using the SAS statistical software (version 8.1, SAS Institute Inc., Cary, NC, USA) [4]. Bacterial cell concentration and *AWCD*_590nm_ differences between “before” and “flushed” samples were compared using Student's *t*-test. To explore the effects of indoor heating on water bacterial community functional metabolic fingerprints, heat map analyses of thirty one different carbon sources utilization was performed using R software. Principle component analysis (PCA) was performed using SPSS Version 16.0 software for Windows (SPSS Inc., Chicago, IL, USA), and the first two PC1 and PC2 were selected. Graphical work was carried out using the Sigma Plot (Version 10.0) software package for Windows.

## 3. Results and Discussion

### 3.1. Water Temperature and Quality

There was a significant difference between the overnight stagnated water and flushed water temperature (*p* < 0.01). The temperatures of overnight stagnated water (“before”) were 16.5 °C, 14 °C, 13.5 °C, 15 °C, and 14.1 °C for Tap 1, Tap 2, Tap 3, Tap 4, and Tap 5, respectively. The temperatures of flushed water (“flushed”) were 4.2 °C, 4.5 °C, 10.1 °C, 8.2 °C, and 7.3 °C for Tap 1, Tap 2, Tap 3, Tap 4, and Tap 5, respectively. However, as shown in Table 2, there were no significant differences in pH, dissolved organic carbon, UV_254_, turbidity, and residual chlorine between water stagnated overnight and flushed water samples (*p* > 0.05) (Table 2).

**Table 2:** Indoor tap water quality of “before” and “flushed” water samples used in this work.

Water Quality Parameters	“Before” Water Samples	“Flushed” Water Samples	T-Text
pH	7.3 ± 0.02	7.2 ± 0.03	NS
Dissolved organic carbon (mg/L)	1.8 ± 0.1	1.9 ± 0.3	NS
UV_254_	0.08 ± 0.0	0.09 ± 0.0	NS
Turbidity (NTU)	0.5 ± 0.0	0.4 ± 0.0	NS
Residual chlorine (mg/L)	0.2 ± 0.0	0.3 ± 0.0	NS

NS indicates no significant difference between “before” and “flushed” water samples (n = 5).

### 3.2. Bacterial Cell Concentration

**Figure 1:**
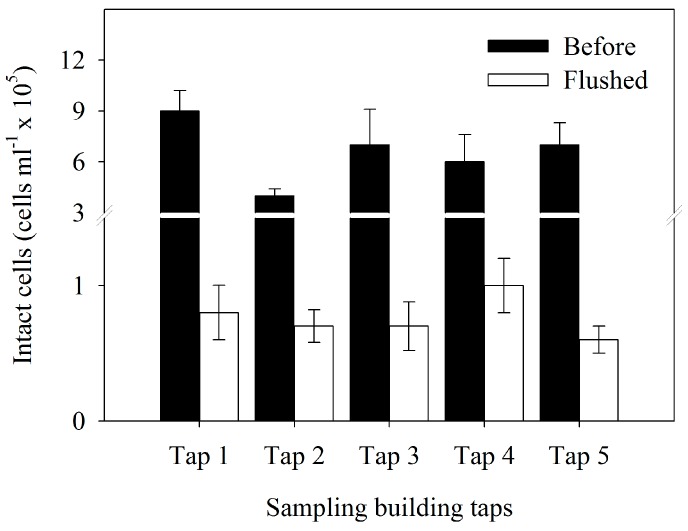
Intact bacterial cell numbers from the different sampling building tap waters before flushing (“before”) and after flushing for 10 min (“flushed”). The data is expressed by as the mean and standard errors (*n* = 3).

Indoor heating can increase the water bacterial cell concentration in water stagnated overnight in building tap pipes. As shown in Figure 1, the highest bacterial cell concentration (9 × 10^5^ cells/mL) was observed in Tap 1 “before” water. After 10 min of flushing, the cell concentration was decreased sharply in Tap 1 “flushed” water (*p* < 0.001). A significant lowest bacterial cell concentration (0.6 × 10^5^ cells/mL) was observed in Tap 5 “flushed” water (*p* < 0.001) (Figure 1).

Univariate analyses were employed for the significant effects in multivariate analysis (Table 3). The significant “flushed” and “tap” values indicate that the bacterial cell concentration varied between “before” and “flushed” water samples. However, there were no significant differences between the five taps (*p* > 0.05) (Table 3).

**Table 3:** Two-way ANOVA of the bacterial cell number and bacterial community functional metabolic profiles of the building tap water from before flushing (“before”) and flushed for 10 min (“flushed”) of Tap 1, Tap 2, Tap 3, Tap 4, and Tap 5.

Bacterial Parameters	Flushed (*F*-Value)	Tap (*F-*Value)	Interaction (*F*-Value)
Bacterial cell number	252.65 ***	12.56 **	6.83 *
*AWCD*_590nm_	156.21 ***	16.92 **	5.61 *
Carbohydrates	58.61 **	1.04NS	0.84NS
Carboxylic acids	35.47 **	6.32 *	5.04 *
Amino acids	14.28 **	4.59 *	1.09NS
Polymers	1.53NS	0.96NS	0.08NS
Phenolic compounds	19.21 **	8.62 **	1.68NS
Amines	66.37 **	0.43NS	1.35NS

*NS*, not significant, *p* > 0.05, ^*^ *p* < 0.05, ^**^ *p* < 0.01, ^***^ *p* < 0.001, the bold font value was not significant.

### 3.3. Bacterial Community Metabolic Profiles

**Figure 2:**
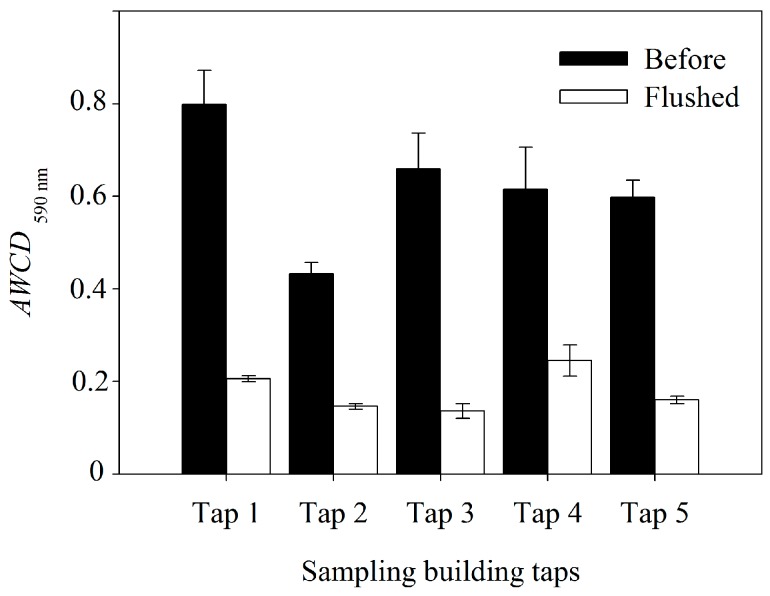
Average Well Color Development (*AWCD*_590nm_) of bacterial functional community in the tap water collected from building taps before flushing (“before”) and flushing for 10 min (“flushed”). The data expressed by the mean and standard errors (*n* = 3).

The building tap pipes water bacterial community metabolic activity (*AWCD*_590nm_) can be significantly improved by the indoor heating process after overnight stagnation (Figure 2).

As shown in Figure 2, the *AWCD*_590nm_ of “before” water samples were significantly higher than that of the “flushed” water samples (*p* < 0.001). The highest *AWCD*_590nm_ (0.798) was found in Tap 1 “before” water, and after 10 min flushing, the *AWCD*_590nm_ decreased to 0.205.

**Figure 3:**
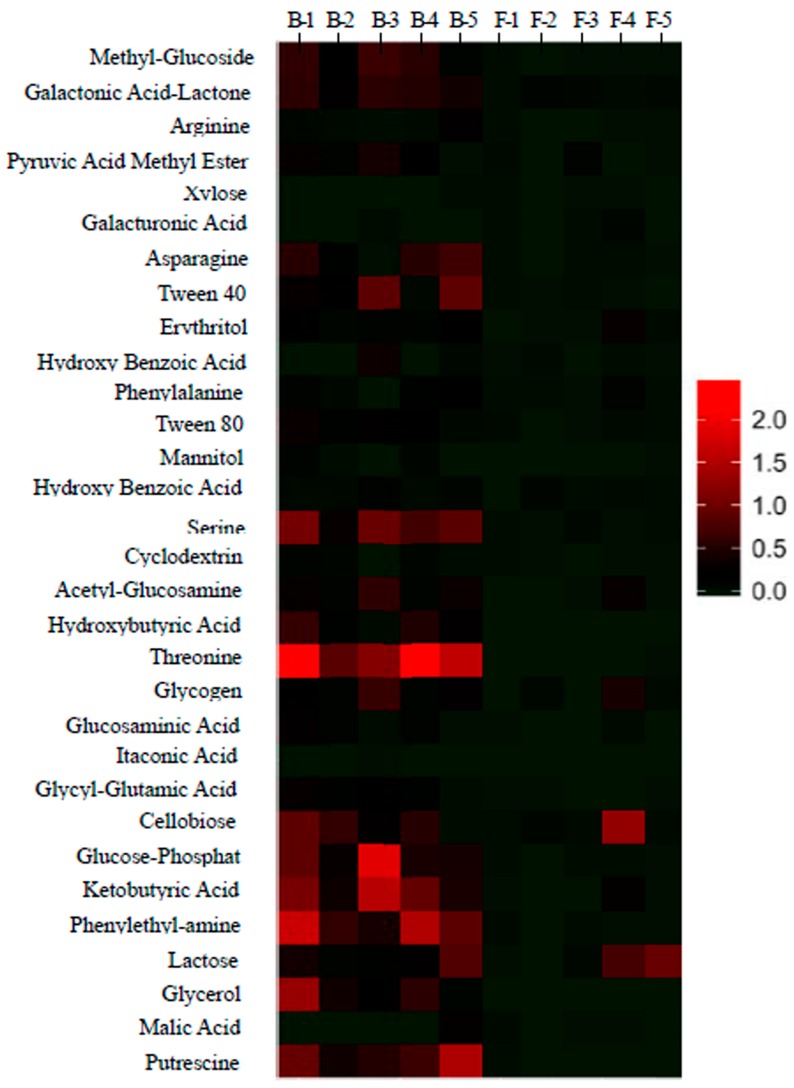
Heat map fingerprints of water bacterial community utilized different sole carbon substrate in the tap water collected from building taps before flushing (Before) and flushing for 10 min (Flushed). B-1, B-2, B-3, B-4, and B-5 represent Tap 1, Tap 2, Tap 3, Tap 4, and Tap 5 of water before flushing, respectively. F-1, F-2, F-3, F-4, and F-5 represent Tap 1, Tap 2, Tap 3, Tap 4, and Tap 5 of flushed water, respectively.

Meanwhile, *AWCD*_590nm_ and different carbon sources utilization varied in both “flushed” and “tap” samples, as indicated by multi analysis of variance (MANOVA) (Table 2). The heat map fingerprint suggested threonine, glucose-phosphate, ketobutyric acid, phenylethvlamine, glycerol, putrescine, and serine were significantly used in “before” water samples (Figure 3).

**Figure 4:**
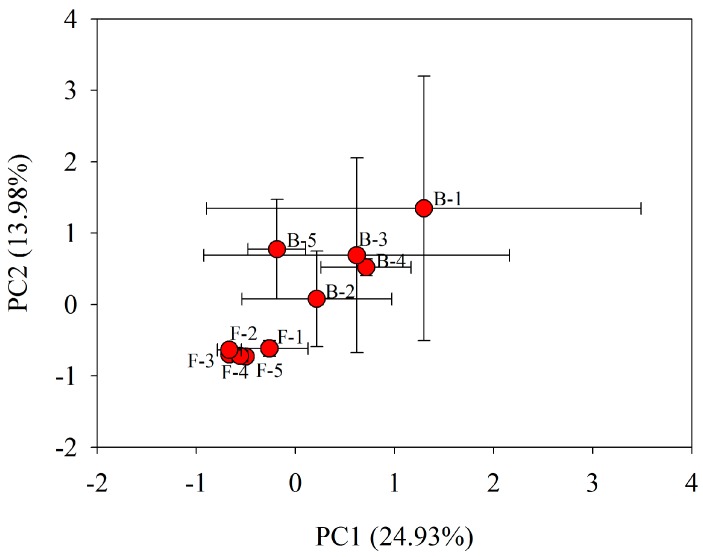
Principal components analysis (PCA) of 31 different sole carbon substrate metabolic profiles of bacterial functional community in the tap water collected from building taps before flushing (“before”) and flushing for 10 min (“flushed”). The data is expressed by the mean and standard errors (*n* = 3). PC1 explains 24.93% of the variance of the data and PC2 explains 13.98% of the variance in the data. B-1, B-2, B-3, B-4, and B-5 represent Tap 1, Tap 2, Tap 3, Tap 4, and Tap 5 of water before flushing, respectively. F-1, F-2, F-3, F-4, and F-5 represent Tap 1, Tap 2, Tap 3, Tap 4, and Tap 5 of flushed water, respectively.

Principle component analyses (PCA) demonstrated that a significant bacterial cell number and community functional metabolic profile discrimination existed between the overnight stagnated water and flushed water (Figure 4). As shown in Figure 4, the first two principles explained 38.91% of the total variance. PC1 and PC2 explained 24.93% and 13.98% of the variance, respectively. It is suggest that the “before” water bacterial community functional diversity is more unstable than that of the “flushed” water samples (Figure 4). Therefore, these data revealed that bacterial community metabolic fingerprints were changed due to the indoor heating process. The water stagnated overnight in the indoor pipes should be treated prior to people’s drinking consumption.

Microbial pollution in drinking water is a major public health concern. Drinking water safety is a hot topic, especially in the development countries. Water born microbial contamination of drinking water should be roundly determined from the drinking water reservoir, drinking water treatment processes to the point of household taps [1,3]. As we all known, the growth of bacterial species harbored in the indoor drinking water taps was regulated by external environmental factors, and the people’s health and drinking water safe was influenced by water quality of indoor drinking water taps. The indoor temperature increased significantly by indoor heating process [12]. However, the mechanism of effect of indoor heating on bacterial regrowth from indoor drinking water taps was vastly not well understood.

In this work, the temperatrue of water stagnated overnight increased significantly, and bacterial quality of tap water was found to deteriorate due to the overnight stagnation. This result is consistent with study conducted by Inkinen *et al.* [23], who determined biofilm formation in an office building during its first year of operation, which showed stagnation increased viable biomass concentrations in the water pipes. Bagh *et al.* [24] also examined the distribution of bacteria in a Danish domestic hot water system, and suggested that heterotrophic plate counts were five-fold higher in the hot water pipe than in the cold water pipe. Lipphaus *et al.* [25] used flow cytometry to determine bacterial concentrations in water sampled from coffee kitchen, bathrooms, and laboratories, and found stagnation could increase bacterial cell numbers in tap water, whereby cell numbers ranged from 1.66 × 10^3^ to 4.31 × 10^5^ for intact cells. The reason most important was higher water temperature enrichment with nutrients that were found in stagnant parts compared with the mains pipe. In the present study, the intact cell numbers ranged from 0.6 × 10^5^ to 9 × 10^5^ cells/mL in the sampled waters, which is higher than that of Lipphaus *et al.* [25]. The most possible explain for this phenomenon was different conditions including water fluent, water quality, pipe materials, biofilm formation and pipeline use times. Water distribution system (WDS) microbial community diversity was also shaped by environmental conditions, pipes materials and using periods [26]. Charisiadis *et al.* [27] studied the spatial and seasonal variability of tap water disinfection by-products within distribution pipe networks; tap water sampling was conducted with high air temperatures ranged between 30–45 °C, and low air temperatures ranged between 0–15 °C. The results suggested tribromomethane showed large seasonal variability in tap water samples. In this work, we found that water bacterial community metabolic characteristics were changed due to the indoor heating, which increased the stagnated water temperature, which in turn mproved the bacterial community activity. Temperature may increase the development of biofilm in pipes [28]. Free-living bacteria can harbered on the surface of biofilm of pipelines. Rakic *et al.* [29] determined the presence of *Legionella pneumophila* in water distribution mains and in consumers’ plumbing systems in the Dalmatian region of Croatia, and the positive correlation was found between the water temperature and *L. pneumophila* contamination. In this work, the material of pipes was galvanized steel, and Lin *et al.* [30] suggested that PVC would be more suitable for use in household pipes, because the microbial community diversity is lesser than that of stainless steel and cast iron pipes [30]. Heinrichs *et al.* [31,32] determined the black fungal biofilms occurring at domestic water taps using the Tag-Encoded FLX Amplicon Pyrosequencing technique, and revealed that the potential biological hazards caused by fungi is low. However, in this work, we did not determine the fungal species. Fungal species can release fungal metabolites into the water and several bacteria can cause odors [33].

Microbiologists and water treatment engineering designers are beginning to collaborate in a new field focused on the microbiology of the water distribution systems and indoor environmental conditions. To our best knowledge, we remain in the very early stages of understanding their relationship, and no literature is available yet on the bacterial community functional profiles in tap water. The results from the present work will facilitate the development of useful control techniques that will ensure safe and high quality tap drinking water. It may be possible to design and develop methods that inhibit the growth bacterial community in order to promote healthier indoor tap drinking water. Although important data were obtained in the present work, further research is needed to clarify the life history, transportation, and biofilm formation of fungal, algal, and virus cells harbored in building indoor pipelines using high-throughput sequencing. Meanwhile, stable isotope probing linking the microbial identity with function, has proved its important role in biodegradation and bioremediation. This technique holds the potential to characterize the microbial species incorporating different substrates in drinking water systems [34,35,36,37,38,39].

## 4. Conclusions

Biological stability of drinking water is related to people’s health. In this work, flow cytometry and BIOLOG techniques were used to reveal the effects of the indoor heating process on building tap pipe water bacterial cell concentrations and community carbon metabolic profiles. The results suggested that the temperature of overnight stagnation water was higher than that of the water after 10 min of flushing. The highest bacterial cell number was observed in water stagnated overnight, which is 5–11 times higher than that of flushed water. Meanwhile, a significantly higher bacterial community metabolic activity was also found in overnight stagnation water samples. Principle component analyses revealed a significant difference in the bacterial community functional metabolic profiles between the water stagnated overnight and flushed water. Serine, threonine, glucose-phosphate, ketobutyric acid, phenylethylamine, glycerol, and putrescine were significantly used by overnight stagnation water samples. The results from this work suggested that stagnated water with higher temperature should be treated before drinking consumption because of bacterial regrowth during the winter season.
